# Noninvasive and Quantitative Assessment of *In Vivo* Fetomaternal Interface Angiogenesis Using RGD-Based Fluorescence

**DOI:** 10.1155/2014/309082

**Published:** 2014-07-10

**Authors:** M. Keramidas, J. Lavaud, F. Sergent, P. Hoffmann, S. Brouillet, J.-J. Feige, J.-L. Coll, N. Alfaidy

**Affiliations:** ^1^Institut National de la Santé et de la Recherche Médicale (INSERM), Unité 823, Institut Albert Bonniot, Rond-point de la Chantourne, 38700 La Tronche, France; ^2^Université Grenoble Alpes, 38041 Grenoble, France; ^3^Commissariat à l'Énergie Atomique et aux Énergies Alternatives, Institut de Recherches en Sciences et Technologies pour le Vivant (iRTSV), Biologie du Cancer et de l'Infection, 17 rue des Martyrs, 38054 Grenoble, France; ^4^Institut National de la Santé et de la Recherche Médicale (INSERM), Unité 1036, Biologie du Cancer et de l'Infection, 17 rue des Martyrs, 38054 Grenoble, France; ^5^CHU de Grenoble, Hôpital Couple Enfant, Département de Génétique et Procréation, Centre d'Aide Médicale à la Procréation, BP 217, 38043 Grenoble, France

## Abstract

Angiogenesis is a key process for proper placental development and for the success of pregnancy. Although numerous *in vitro* methods have been developed for the assessment of this process, relatively few reliable *in vivo* methods are available to evaluate this activity throughout gestation. Here we report an *in vivo* technique that specifically measures placental neovascularization. The technique is based on the measurement of a fluorescent alpha *v* beta 3 (*α*_*v*_ *β*_3_) integrin-targeting molecule called Angiolone-Alexa-Fluor 700. The *α*_*v*_ *β*_3_ integrin is highly expressed by endothelial cells during the neovascularization and by trophoblast cells during their invasion of the maternal decidua. Angiolone was injected to gravid mice at 6.5 and 11.5 days post coitus (dpc). The fluorescence was analyzed one day later at 7.5 and 12.5 dpc, respectively. We demonstrated that (i) Angiolone targets *α*_*v*_ *β*_3_ protein in the placenta with a strong specificity, (ii) this technique is quantitative as the measurement was correlated to the increase of the placental size observed with increasing gestational age, and (iii) information on the outcome is possible, as abnormal placentation could be detected early on during gestation. In conclusion, we report the validation of a new noninvasive and quantitative method to assess the placental angiogenic activity, *in vivo*.

## 1. Introduction

The placenta is a well-organized and highly vascularized organ [1]. The placental vascular network is composed of an extravillous and an intravillous vascular networks, also called the fetomaternal interface (FI), and the fetal vascular system, respectively. Intravillous neovascularization starts early on during gestation and is followed by an active angiogenesis throughout the first trimester of pregnancy, allowing the prompt growth and branching of the placental vessels [1, 2]. The establishment of the FI circulation (FIC) depends on the complex process of trophoblast differentiation during the first trimester of pregnancy [3, 4]. At around 10–12 wg, trophoblast cells at the tip of the villi become invasive as they differentiate into extravillous trophoblasts (EVT). These EVTs migrate and invade both the decidua and the maternal spiral arteries. The invasion of maternal spiral arteries leads to the remodeling of these vessels from high to low resistance vessels [5].

Both the FIC and the intravillous angiogenic processes are known to be tightly controlled by pro- and antiangiogenic factors [6]. Among proteins that appear to accompany the process of placental angiogenesis and the establishment of the FIC are the integrins. These are a family of glycoproteins that participate in a number of placental functions, including cell adhesion, migration, and invasion [5, 7, 8]. Integrins comprise noncovalently bound *α* and *β* subunits that participate in cell-to-cell and cell-to-substratum adhesion [9]. In the process of the establishment of FIC, four combinations of integrins appear to play important roles. These include *α*_6_ *β*_4_, *α*_5_ *β*_1_, *α*_1_ *β*_1_, and *α*_*v*_ *β*_3_. During their invasion, EVTs become *α*_6_ *β*_4_ integrin-negative and *α*_5_ *β*_1_-, *α*_1_ *β*_1_- and *α*_*v*_ *β*_3_ integrin-positive [10]. Disturbance of the expression of some of these integrins at the surface of EVTs is associated with pregnancy disorders such as preeclampsia [5, 11]. In this disease differentiating/invading trophoblast cells retain expression of *α*_*v*_ *β*_6_ [12, 13] and fail to upregulate *α*_*v*_ *β*_3_. The *α*_*v*_ *β*_3_ is an integrin that displays increased expression levels on the surface of angiogenic endothelial cells as compared with quiescent endothelial cells and was reported as a useful tool to estimate the levels of the neovascularization in a given organ [14, 15]. Previous reports in the literature have shown that *α*_*v*_ and *β*_3_ proteins are highly expressed in mouse placenta with specific localizations to the endothelial and to trophoblast cells present at the fetomaternal interface [16, 17].

Although numerous* in vitro *methods have been developed for the assessment of placental angiogenic activity, relatively few reliable and quantitative methods are available* in vivo* to assess this activity at the fetomaternal interface [18]. The* in vivo* approaches used so far are based on microultrasound analyses and color Doppler blood flow visualization. The disadvantage of these techniques remains their nonquantitative aspect.

Using a 3D optical imaging of fluorescent probes imaging, we have recently described a noninvasive and quantitative assessment of* in vivo* angiogenesis of subcutaneous sponges [19]. The probe used in this study is Angiolone-Alexa-Fluor 700, a fluorescent molecule that targets the *α*_*v*_ *β*_3_ integrin, allowing quantitative determinations of the angiogenic activity,* in vivo*.

Angiolone-Alexa-Fluor 700 is a cyclic pentapeptide presenting the arginine-glycine-aspartic acid (RGD) sequence known to target the *α*_*v*_ *β*_3_ integrin [20]. The tetrameric cRGD-containing peptide, RAFT-c-(RGDfK-)4, was generated by covalently linking four peptides (-cRGDfK-) to the cyclic decapeptide platform “regioselectively addressable functionalized template (RAFT)” [21]. AlexaFluor 700 fluorescent dye was linked to the Angiolone to convert this reagent into an optical imaging probe able to target *α*_*v*_ *β*_3_-expressing cells [22–24].

To measure the accumulation of fluorescence in the vascularized placentas with sufficient precision, we used the previously described 2D Hamamatsu system and continuous-wave fluorescence diffuse optical tomography (fDOT) optical imaging system [23, 24]. This assay is based on the use of fluorescent* in vivo* labeling of the neoformed blood capillaries with Angiolone-Alexa-Fluor 700 and whole body small animal examination with a 2D system and abdominal area with fDOT [23, 24].

Using this technology, we report here the validation of a new noninvasive and quantitative method to assess placental angiogenic activity during gestation in the gravid mouse. Gravid mice were assessed at 7.5 and 12.5 dpc, two main time points that represent placental angiogenesis and establishment of the fetomaternal circulation, respectively.

## 2. Materials and Methods

### 2.1. Animal Experiments

**Figure 1:**
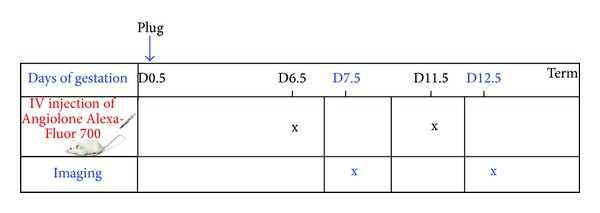
Protocol of gravid mouse treatment. Angiolone-Alexa-Fluor 700 was injected one day before imaging at days 6.5 and 11.5 dpc. Mice were analyzed at days 7.5 and 1.25 dpc, respectively.

Three-month-old pregnant female OF-1 mice were obtained from Charles River Laboratories (Les Oncins, France). All animal studies were approved by the institutional guidelines and by the European Community for the Use of Experimental Animals. Gravid female OF-1 mice were obtained by in-house mating. The date of the presence of a vaginal plug was taken as day 0.5 post coitus (dpc). The gravid females were maintained in the animal facility. They were injected at day 6.5 or day 11.5 dpc of gestation, and after imaging they were sacrificed at 7.5 or 12.5 dpc via a lethal injection of Doletal (Figure 1). These gestational ages correspond to the peak of angiogenic processes in the placenta. At least three mice were used for each gestational age examined. This group of animals was only used for imaging. To assess the level of expression of the complex *α*_*v*_ *β*_3_ in the placenta, we used another set of gravid mice that were sacrificed at 10.5, 14.5, and 17.5 dpc. This group of animals was used as a control group and was not injected by Angiolone-Alexa-Fluor. The choice of the gestational dates to study the expression of *α*_*v*_ *β*_3_ in the placenta was based on the fact that 10.5 dpc corresponds to the placental angiogenic peak and represents the first trimester of pregnancy in women, 14.5 dpc corresponds to the second trimester, and 17.5 dpc will represent the third trimester.

### 2.2. Fluorescence *In Vivo* Imaging

For fluorescence imaging, 200 *μ*L Angiolone-Alexa-Fluor 700 (50 *μ*M) (Fluoptics, Grenoble, France) was injected into the mouse tail vein. For reflectance imaging, mice were illuminated with 660-nm light-emitting diodes equipped with interference filters and fluorescence images, as well as black and white pictures, which were acquired by a back-thinned charge-coupled device (CCD) camera at −80°C (ORCAII-BT-512G; Hamamatsu, Massy, France) and fitted with a high-pass RG 9 filter (Schott, Clichy, France) [22].

**Figure 2:**
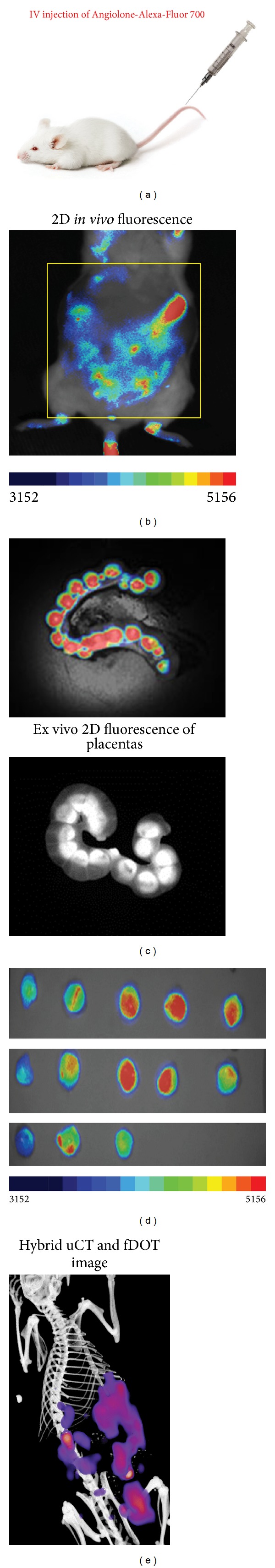
Imaging sequence performed for the gravid mice angiogenesis assay. Panel (a): intravenous injection of Angiolone-Alexa-Fluor 700 on day 6.5 or 11.5 dpc. Panel (b): 2D* in vivo* fluorescence. Vascularization was imaged using the fDOT2D system. Panel (c): 2D fluorescence images in the uterine horns. Panel (d): 2D fluorescence images in isolated placentas. Panel (e): a hybrid image of placentas in an anatomical context. The scale is provided in arbitrary unit because the fluorescence produces relative values unless a standard calibration has been performed.

Three-dimensional fluorescence acquisition and quantification were performed 24 h after injection with the continuous-wave fDOT system previously described by Koenig et al. [23, 24]. fDOT consists of a 690-nm laser source, a CCD camera, and a set of filters. The light source is a 35-mW compact laser diode (Power technology) equipped with a bandpass interference filter (Melles Griot 685AF30OD6). The emitted fluorescence is filtered by two 700-nm high-pass colored glass filters (Schott RG9 OD5) placed in front of a NIR-sensitive CCD camera (Hamamatsu ORCA AG) mounted with a f/15-mm objective (Schneider Kreutznach). The excitation sources described a regular 26 × 30-mm spaced grid over the abdominal area of the mouse, were the embryos are present. Two scans were successively performed for fluorescence and diffusion. The exposure time was automatically computed at each laser position to use the entire dynamic range of the camera. The two stacks of diffusion and fluorescence images were analyzed by the reconstruction algorithm to generate a 3D image. Three-dimensional reconstruction was performed as described previously [26]. fDOT principle lies in the ability to both reconstruct fluorescence even in highly heterogeneous attenuating media and handle complex geometries. The results are presented as a 3D view of the reconstructed area. The reconstructed area is a volume meshed with a 2-mm sample rate in the *x* and *y* directions and 1 mm in the *z* direction (depth) that yields a size of approximately 8 × 10 × 15 voxels and may vary slightly depending on animal thickness.  Figure 2 presents the reconstructed fluorescence in *z* cross-sections. The cross-sections are presented from bottom to top for *z* = 0 (ventral side) and *z* = 15 (dorsal side). The superimposition of the reconstructed volumes viewed as a smooth interpolation perspective and positioned on top of the white-light image of the animal was allowed for the generation of the final image. The procedure time on a 3-GHz intel Xeon was 10 min to reconstruct the fluorescence distribution. Each fluorescence reconstruction is presented with the same color scale to allow for visual comparison.

### 2.3. Scanner *In Vivo* Imaging

To visualize the whole animal body, we performed a medium resolution microCT (VivaCT 40 Scanco Medical) with a 42 *μ*m isotropic voxel size, a voltage of 45 kV, and a current of 114 mA. The 3D fluorescence is merged with the mice's skeleton in order to replace the fluorescent signal in an anatomical context.

### 2.4. Statistical Analysis

All data are expressed as mean ± SEM. Statistical comparisons were made using *t*-test analysis. Calculations were performed using SigmaStat (Jandel Scientific Software, San Rafael, CA).

## 3. Results

### 3.1. Visualization of the Placental Angiogenic Activity in Gravid Mice

In the gravid mice, the labeling of angiogenesis was tracked during 24 h after injection of Angiolone-Alexa-Fluor 700 (Figure 2). The probe produced a positive staining in the treated animals throughout the 24 h following the injection. A semiquantitative measurement allowed us to determine that the best signal to noise ratio in the placenta was obtained as early as 24 h after injection of the probe. We, thus, imaged the mice 24 h after injection in the following experiments.

Angiogenic activity in the gravid mice was evaluated in three systems; the whole animal, the dissected gravid horns, and the isolated individual placentas. The* in vivo* level allowed the visualization and the quantification of placental angiogenesis with a specific signal emanating from the intrauterine zone (panel (b)). Dissection of the gravid horns allowed precise localization of the active angiogenic sites within the horn (panel (c)). The strongest angiogenic activity was observed in placentas localized at the base of the horn compared to those localize at its end. When considering isolated placentas, the angiogenic activity was concentrated at the fetomaternal interface (Panel (d)). This observation was confirmed in isolated placentas. Panel (e) shows a hybrid image that reports 3D fluorescent signal from the placentas in an anatomical context.

### 3.2. Comparison of Placental Angiogenic Activities during Early Pregnancy

**Figure 3:**
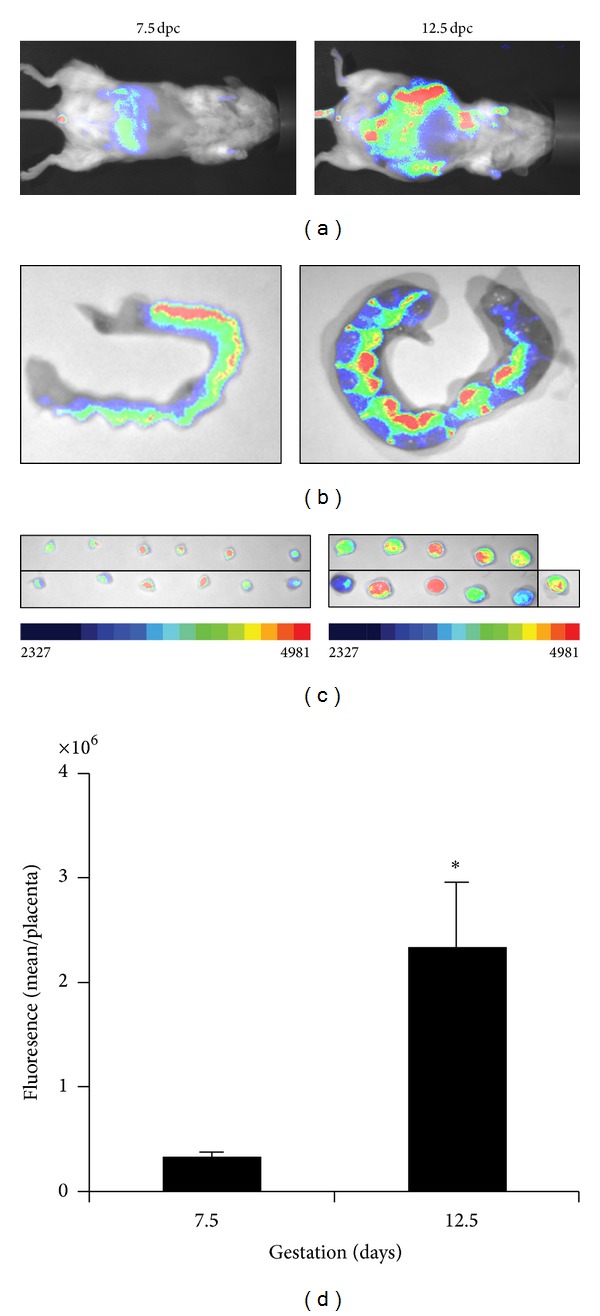
Quantification and comparison of placental angiogenic activity in gravid mice at 7.5 and 12.5 dpc. Panel (a) compares fluorescence emanating from the two gravid mice at 7.5 dpc and 12.5 dpc. Panel (b) compares the fluorescence at the level of dissected uterus. Panel (c) compares the fluorescence in the placentas dissected from the 7.5 and 12.5 dpc horns. Panel (d) reports the comparison of the levels of the fluorescence in the placentas. Fluorescence was measured in each individual placenta and reported as mean fluorescence per placenta.

During mouse gestation, neovascularization starts early on during gestation with the highest activity occurring around 12.5 dpc. Using our imaging system, we compared the levels of the placental angiogenic activity in isolated placentas at 7.5 and 12.5 dpc.  Figure 3 shows comparisons of the angiogenic activities at the animal, horn, and placentas levels. Angiogenic activity was detected as early as 7.5 dpc (Figures 3(a), 3(b), and 3(c)) and significantly increased at 12.5 dpc. Differences in the signals were observed at the three levels. Quantification of the fluorescence in isolated placentas shows that the angiogenic activity at 12.5 dpc was 6 times higher than the one measured at 7.5 dpc (Figure 3(d)).

### 3.3. Detection of Abnormal Placental Angiogenic Activity during Gestation

**Figure 4:**
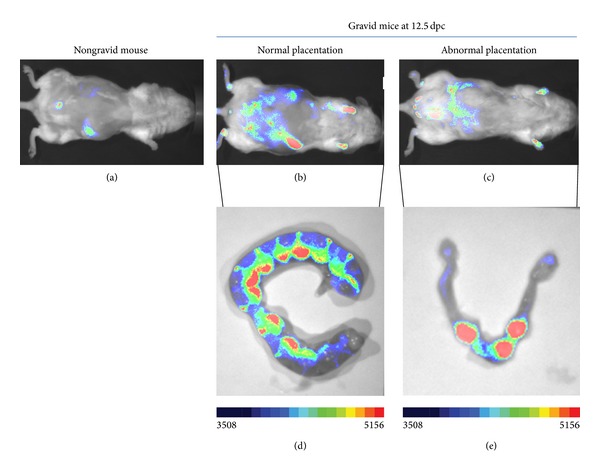
Illustration of detection of abnormal placentation using the proposed technique. Photographs in Panels (a), (b), and (c) show three mice, nongravid mouse, 12.5 dpc gravid mouse with normal placentation, and a 12.5 dpc gravid mouse with abnormal placentation, respectively. Panels (d) and (e) compare the horns dissected from the mice undergoing normal and abnormal placentations, respectively.

In the series of the gravid mice analyzed in this study we came across a gravid mouse that exhibited low fluorescence signal at 12.5 dpc (Figure 4(c)) as compared to age-matched control gravid mice (Figure 4(b)). Dissection of its horns illustrated in Figure 4(e) showed horns with reduced size compared to the horn of mice at 12.5 dpc (Figure 4(d)). Furthermore, the gravid mouse had only three placentas confirming the reduced fluorescence observed in whole animal analysis. These data reveal the potential use of the noninvasive 2D fluorescence to detect abnormal placentation during gestation.

## 4. Discussion

The placenta is by far the most angiogenic organ with the highest angiogenic activity occurring in early gestation [27]. A normal angiogenic activity during this period is the key to the success of pregnancy. Hence, a setup of a technique that allows the assessment of this process is of great interest. Using 2D and 3D optical imaging, we established a reproducible method to measure placental angiogenesis in a noninvasive manner during mouse gestation. To our knowledge, this is the first report of a direct and quantitative way to assess physiological and pathological placental angiogenesis. The specificity of this technique was verified by the intensification of the measured signal between 7.5 and 12.5 dpc, which correlates with the established increase in the intravillous and the FC angiogenic activities. More importantly, we demonstrated that this technique could be informative on abnormal placentation through defects in the angiogenic activity.

To date, most experimental placental imaging techniques are being used in post mortem specimens and consist of vascular corrosion casting and microcomputed tomography [28–30]. These techniques investigate normal villous vasculogenesis and angiogenesis, by (i) applications of corrosion casts and observations under a scanning electron microscope, (ii) injection of contrast medium, or (iii) using a classical microscope and manual 3D reconstructions of paraffin sections. Though these techniques have improved over the years, they still provide qualitative evaluation of placental phenotypes [18, 28–30]. The advantage of the proposed technique is its specificity to target placental neovascularization, a key aspect of placental development. More importantly, this technique might help in (i) the diagnoses of placental abnormalities, (ii) the evaluation of invasive placenta, such as placenta accreta, and (iii) the assessment of placental perfusion.

The proposed technique was based on targeting of *α*_*v*_ *β*_3_, a key integrin protein of placental angiogenesis. The physiological importance of integrins during angiogenesis has been most extensively studied in the case of the *α*_*v*_ integrins. Antagonists of *α*_*v*_ *β*_3_ and *α*_*v*_ *β*_5_ integrins block growth-factor- and tumor-induced angiogenesis in multiple animal models [3, 31]. Furthermore, recent data from clinical trials suggest that antagonists of *α*_*v*_ *β*_3_ and/or *α*_*v*_ *β*_5_ may have a clinical benefit in humans with solid tumors [32–34].

Beyond their importance as molecular tools to specifically allow targeting sites of neovascularization, integrins have been reported to play major roles in placentation processes and their deregulation is associated with placental pathologies such as preeclampsia, the most threatening pathology of human pregnancy [5, 13]. Interestingly preeclamptic placentas have been shown to express low levels of *α*_*v*_ *β*_3_ integrins [5], suggesting that this integrin may be important in proper placental progression and that this complex might provide a target for therapeutic intervention [35]. Moreover the use of this integrin complex as a target in the proposed technique can be informative on the outcome of an ongoing abnormal pregnancy.

Because it was impossible to prospectively study placental angiogenic processes in humans, we used the murine placenta to test this new technique. While some of the gross anatomy and physiology of mouse and human placentas are different, these two species show a similar haemochorial type of placentation and considerable histological and mechanistic similarities in placental development [36–38]. Hence, studies of placentation in mice are likely to yield new insights into therapies and into the use of new imaging techniques in human pregnancy. Validation of this technique in primates should lead to its better characterization in the view to its transfer to human pregnancy.
